# A machine learning-based model for assessing community-acquired pneumonia severity using routine blood tests

**DOI:** 10.3389/fcimb.2025.1605502

**Published:** 2026-01-12

**Authors:** Chao Guan, Fei Chen, Yunxiao Song, Ying Huang, Ying Zhou, Zhiliang Wang, Jie Cheng

**Affiliations:** 1 Department of Respiratory Medicine, Shanghai Xuhui Central Hospital, Fudan University, Shanghai, China; 2 Department of General Surgery, Putuo People’s Hospital, School of Medicine, Tongji University, Shanghai, China; 3 Department of Clinical Laboratory, Shanghai Xuhui Central Hospital, Fudan University, Shanghai, China; 4 Department of General Medicine, Shanghai Xuhui Central Hospital, Fudan University, Shanghai, China; 5 Department of Urinary Surgery, Shanghai Xuhui Central Hospital, Fudan University, Shanghai, China

**Keywords:** community-acquired pneumonia, routine blood indicators, machine learning, severity, random forest

## Abstract

**Background:**

The choice of first-line therapy for community-acquired pneumonia (CAP) depends on disease severity. However, quickly and accurately differentiating mild from severe CAP patients remains challenging. This study aims to evaluate the performance of machine learning-based diagnostic models employing routine blood indicators to distinguish CAP severity.

**Methods:**

A multicenter, retrospective, case–control study conducted at Xuhui Central Hospital (Discovery cohort), and Putuo People’s Hospital (Validation cohort), from January 2016 to January 2024. Patients were further classified into mild or severe CAP according to the IDSA/ATS criteria. Routine blood tests were performed with an automatic blood cell analyzer. Twelve machine learning-based diagnostic models were developed from routine blood indicators for differentiating between mild and severe CAP.

**Results:**

A total of 3,127 (1,612 mild, 1,615 severe) and 2,087 participants (1,072 mild, 1,015 severe) were included in the discovery and validation cohorts, respectively. Of the 12 models developed, the random forest (RF) model showed the best performance with 9 routine blood indicators. In the discovery cohort, the model achieved an AUC of 0.95, an AUPRC of 0.94, a positive predictive value of 0.89, a negative predictive value of 0.88, an accuracy of 0.89, and an F1 score of 0.89, while in the validation cohort, it demonstrated similar performance, with values of 0.95, 0.94, 0.88, 0.87, 0.88, and 0.87, respectively. Decision curve analysis confirmed consistent net benefits from the model across all threshold probabilities. The RF model was integrated into a web application for clinical use.

**Conclusion:**

We successfully developed a nine-feature RF model with promising value for differentiating mild from severe CAP patients.

## Introduction

Community-acquired pneumonia (CAP) is a disease characterized by inflammation of the pulmonary tissue that can lead to severe complications such as sepsis, acute respiratory distress syndrome, or death, making it a leading cause of mortality worldwide (The top 10 causes of death; Aliberti et al., 2021). First-line therapy for CAP differs primarily according to disease severity (Musher and Thorner, 2014; Vaughn et al., 2024). Inappropriate treatment for CAP outpatients or delayed inpatient admission to intensive care units (ICUs) has been associated with increased mortality (Ewig et al., 2009; Liu et al., 2016). Therefore, early identification and management of patients with existing or potentially severe disease is essential, as it would help identify those who may benefit from continuous monitoring to detect complications and reduce mortality.

Risk stratification of patients with CAP is one of the most critical challenges for clinicians, as it facilitates early diagnosis and the selection of appropriate treatments. There is an urgent demand for prognostic tools to better classify patients according to the required level of care (Menéndez et al., 2009). Widely recommended tools, such as CURB-65, CRB-65, and the pneumonia severity index, are commonly used to predict 30-day mortality in CAP patients (Chalmers et al., 2010). However, their performance in assessing the need for ICU admission is limited. While clinical criteria and severity scores can provide admission guidance, the subjective nature of the symptoms of CAP and delays in blood biochemical testing can hinder timely and accurate evaluation (Zhu et al., 2024). Thus, a simple, noninvasive method for rapidly distinguishing between mild and severe CAP is essential for guiding treatment and halting disease progression.

Blood biomarkers present a promising alternative or complement to clinical scoring systems, offering the potential for more precise prognoses and better-tailored treatments. The complete blood count, one of the most frequently performed tests in clinical practice, can provide valuable insights into a patient’s immune response and inflammation levels (Wen et al., 2022). Models based on routine blood tests have been employed to predict the risk of death in emergency medical admissions (Faisal et al., 2017; Ellis et al., 2020) and to predict the outcomes of patients with various diseases (Mao et al., 2023; Osman et al., 2024). Several previous studies have also reported significant associations between routine blood test parameters and the presence or severity of pneumonia (Zhang et al., 2023; Yeşildağ et al., 2024; Ari et al., 2025; Miao et al., 2025). For example, Yeşildağ et al. (2024) reported that the white blood cell count (WBC)/mean platelet volume (MPV) and C-reactive protein (CRP)/MPV ratios were significantly elevated in patients with pneumonia requiring hospitalization. Among these markers, the CRP/MPV ratio demonstrated superior diagnostic performance in identifying patients needing inpatient care. Given the clinical importance of determining whether patients with CAP require hospitalization or can be managed on an outpatient basis, these easily obtainable complete blood count may provide a practical and rapid tool for early triage. Their timely assessment could facilitate prompt and appropriate treatment decisions, potentially reducing morbidity and mortality in pneumonia patients.

Although several prior studies have highlighted potential associations between routine blood biomarkers and the diagnosis or severity assessment of CAP, many of these investigations were limited by small sample sizes and lacked external clinical validation, thereby restricting their applicability to broader patient populations. Therefore, the aim of this study is to develop and externally validate a machine learning model based on routine blood test parameters, using a larger sample size, to enable rapid and accurate assessment of disease severity in patients with CAP.

## Materials and methods

### Patients and study design

This multicenter, retrospective, case–control study was conducted at Xuhui Central Hospital, Fudan University, and Putuo People’s Hospital, School of Medicine, Tongji University, from January 2016 to January 2024. The study was approved by the Ethics Committee of Xuhui Central Hospital (2024–003), Fudan University, in accordance with the Declaration of Helsinki. Informed consent was obtained from all participants.

A total of 5,214 patients with CAP were consecutively enrolled in the study, including 3,127 individuals in the discovery cohort from Xuhui Central Hospital and 2,087 individuals in the validation cohort from Putuo People’s Hospital. Demographic characteristics and clinical information were gathered from the electronic medical records system of the hospital.

### Inclusion and exclusion criteria

The inclusion criteria (Farhat et al., 2024; Zhu et al., 2024) and exclusion criteria (Glöckner et al., 2022; Farhat et al., 2024; Zhu et al., 2024) were applied as previously described and are summarized as follows.

The inclusion criteria were as follows: community-acquired infection; age ≥18 years; new or worsening lung infiltrates observed on chest imaging; at least one of the following: fever (temperature ≥38.3 °C), cough, purulent sputum production, or focal chest signs on auscultation; and routine blood tests results obtained at the time of presentation.

**Figure 1:**
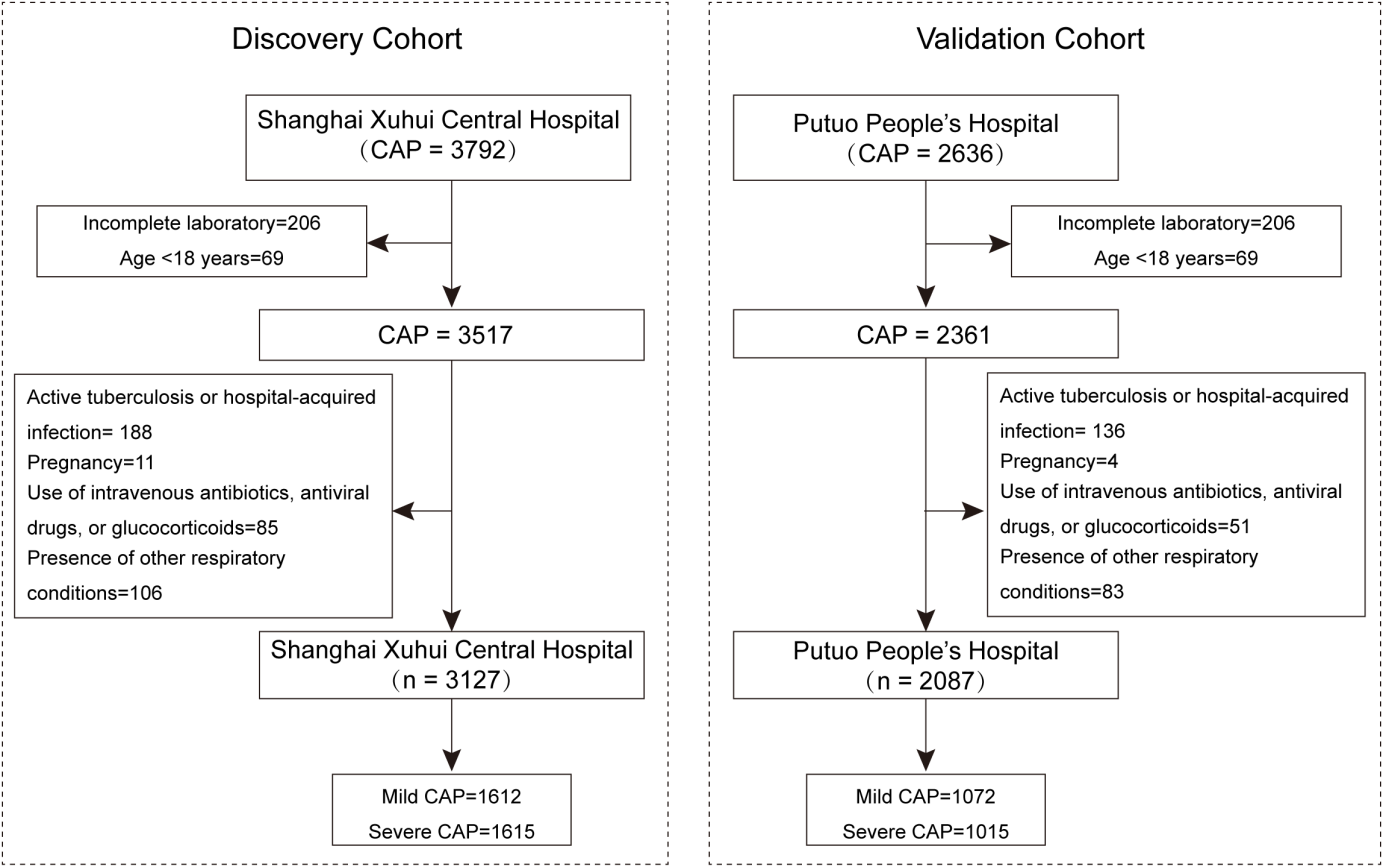
Flow diagram illustrating the inclusion and exclusion process of the study cohort.

The exclusion criteria were as follows: acquired or therapy-induced immunodeficiency; active tuberculosis or hospital-acquired infection; pregnancy; use of intravenous antibiotics, antiviral drugs, or glucocorticoids within the past two weeks; and the presence of other respiratory conditions, such as lung cancer, chronic obstructive pulmonary disease, or bronchiectasis. **Figure 1** illustrates the inclusion and exclusion process of the study cohort.

### CAP severity

On the basis of the “Diagnosis and Treatment of Adults with Community-acquired Pneumonia” guidelines and the Infectious Diseases Society of America/American Thoracic Society (IDSA/ATS) guidelines, patients with CAP in both model cohorts were classified into mild and severe categories (Metlay et al., 2019). Severe CAP is defined by the presence of either one major criterion or three or more minor criteria. Major criteria: (1) Respiratory failure requiring invasive mechanical ventilation; (2) Septic shock requiring vasopressor support. Minor criteria: (1) Respiratory rate ≥ 30 breaths/min; (2) PaO_2_/FiO_2_ ratio ≤ 250; (3) Multilobar infiltrates; (4) Confusion or disorientation; (5) Blood urea nitrogen ≥ 20 mg/dL; (6) white blood cell count < 4,000/mm³; (7) platelet count < 100,000/mm³; (8) core temperature < 36 °C; (9) Hypotension requiring aggressive fluid resuscitation (systolic blood pressure < 90 mmHg).

All patients with CAP were categorized into five risk classes (I–V) based on the Pneumonia Severity Index (PSI) score (Fine et al., 1997).

### Laboratory examinations

Laboratory tests were conducted in duplicate at the Department of Clinical Laboratory, Shanghai Xuhui Central Hospital and Putuo People’s Hospital, as previously described (Cheng et al., 2021), and are summarized as follows.

Blood samples were collected from each patient prior to the administration of any medications. A 2 mL blood sample was drawn using standard venipuncture in the antecubital fossa and collected in an ethylenediaminetetraacetic acid tube. Routine blood tests were performed with a Mindray BC-series automatic blood cell analyzer (Shenzhen, China) within 30 minutes of blood collection. Twenty-four variables were recorded from the routine blood tests: neutrophil count, neutrophil%, red blood count (RBC), platelecrit (PCT), platelet count (PLT), platelet distribution width (PDW), hemoglobin (HG), eosinophil count, eosinophil%, basophil count, basophil%, MPV, lymphocyte count, lymphocyte%, hematocrit (HCT), monocyte count, monocyte%, platelet large cell ratio (PLCR), WBC, red blood cell distribution width-standard deviation (RBWSD), red blood cell distribution width-coefficient of variation (RBWCV), mean corpuscular volume (MCV), mean corpuscular hemoglobin concentration (MCHC), and mean corpuscular hemoglobin (MCH).

### Model development and comparison

Models were built from the 24 features from the routine blood tests via 12 machine learning algorithms, including AdaBoost, decision tree (DT), light gradient boosting (LGB), k-nearest neighbors (KNN), generalized linear model (GLM), logistic regression (LR), random forest (RF), gradient boosting (GB), support vector machine (SVM), extreme gradient boosting (XGB), naïve Bayes, and TabNet. The models were optimized through grid search and manual tuning.

The average area under the receiver operating characteristic (ROC) curve (AUC) from fivefold cross-validation was used to determine the optimal hyperparameters. Model performance was assessed using metrics such as the AUC, sensitivity, specificity, positive predictive value (PPV), negative predictive value (NPV), accuracy, and F1 score. The DeLong nonparametric test was used to compare AUC values across the models and identify the best-performing model. The cut-off thresholds used in the ROC curve analyses were determined based on the Youden Index, which identifies the point on the ROC curve that maximizes the sum of sensitivity and specificity (i.e., sensitivity + specificity-1).

The model parameters were as follows (Cheng et al., 2025): XGB (objective=‘multi:softmax’, colsample_bytree=1.0, learning_rate=0.2, max_depth=7, n_estimators=100, subsample=0.8), RF (n_estimators=100, max_depth=10, random_state=42), DT (max_depth=10, random_state=42), SVM (n_splits=5, shuffle=True, random_state=42), GNB (learning_rate=0.1, max_depth=7, min_samples_leaf=1, min_samples_split=2, n_estimators=50, subsample=1.0), TabNet (max_epochs=100, patience=20, batch_size=32, virtual_batch_size=128, num_workers=0, drop_last=False, weights=1), KNN (n_neighbors=5), LGB (max_depth=10, learning_rate=0.11, n_estimators=100, random_state=42), LR (max_iter=1000, random_state=42), GLM (family=sm.families.Binomial), DT(‘criterion’: ‘entropy’, ‘max_depth’: 5, ‘min_samples_leaf’: 4, ‘min_samples_split’: 2), and GB (learning_rate’: 0.1, ‘max_depth’: 7, ‘max_features’: ‘log2’, ‘min_samples_leaf’: 1, ‘min_samples_split’: 2, ‘n_estimators’: 50, ‘subsample’: 1.0).

### Feature reduction and model determination

Feature reduction helps eliminate noise, streamline models, and enhance their interpretability. In our study, SHapley Additive exPlanations (SHAP) was employed to quantify the importance of each feature in the two-class machine learning model. Features were ranked based on their mean absolute SHAP values in descending order.

To determine the optimal feature subset, we progressively reduced the number of features from the full set (24 variables) down to a single feature, sequentially evaluating model performance at each step. For each reduced feature set, a new model was trained, and its AUC was compared to that of the full-feature model using the DeLong nonparametric test. A p-value < 0.05 was considered statistically significant. The smallest feature subset that maintained comparable predictive performance (i.e., no significant drop in AUC) was selected as the optimal feature set for the final model. This model was then evaluated in the validation cohort to assess its generalizability.

### Web-based model deployment

To facilitate clinical application, the final model was implemented as a web application using the Streamlit Python framework. In the application, users can input the feature values, and the model will provide the probability for each category along with the most likely category.

### Sample size calculation

This calculation was performed under the assumption of a balanced case–control design. To estimate the minimum required sample size, we conducted a power analysis using PASS software. The input parameters were set as follows: expected sensitivity and specificity of 0.80, each with a margin of error of 0.05, and a two-sided significance level (α) of 0.05. Based on these criteria, the calculated minimum sample size was 246 participants per group to achieve sufficient statistical power for evaluating the diagnostic performance of the biomarkers.

### Statistical analysis

Normality was assessed with the Shapiro–Wilk test. Statistical differences between groups were analyzed with appropriate tests: the independent-samples Student’s t test for normally distributed continuous variables, the Kruskal–Wallis test for nonnormally distributed continuous variables, and the chi–square test for categorical variables. The relationships among the variables were assessed via Pearson analysis. Continuous variables are expressed as the means ± SDs, and categorical variables are reported as frequencies and percentages. Data analysis and graphing were performed in GraphPad Prism Software version 9.0 (GraphPad Software, Inc., San Diego, CA, USA), SPSS software (version 19.0; SPSS Inc., Chicago, IL, USA), R 4.0.2 (R Core Team), Python (version 3.11), and PyCharm (version 2023.3.5). A p value < 0.05 was considered to indicate statistical significance.

## Results

### Baseline characteristics of CAP patients

A total of 5,214 patients with CAP were enrolled in the study, comprising 2,684 mild patients and 2,530 severe patients. There were no significant differences (P>0.05) between the mild and severe groups across key indicators. Significant differences were observed in all routine laboratory indicators between mild and severe CAP patients (P < 0.05) except the eosinophil count and basophil count (P > 0.05), as shown in **Supplementary Table S1**.

A total of 3,217 patients were enrolled in the discovery cohort, consisting of 1,612 patients with mild disease and 1,615 with severe disease. There were also no significant differences (P>0.05) between the mild and severe groups across these indicators. In this cohort, significant differences were observed in all routine laboratory indicators between mild and severe patients (P < 0.05) except for monocyte percentage, eosinophil count, and basophil count (P > 0.05). These findings are consistent with the results observed in the entire cohort, as detailed in **Supplementary Table S2**.

The validation cohort, consisting of 2,087 participants (1,072 with mild disease and 1,015 with severe disease), was recruited. Similar to the findings in the discovery cohort, significant differences in all routine laboratory indicators were found between mild and severe patients (P < 0.05) except the eosinophil count and basophil count (P > 0.05), which is also in line with the findings from the whole cohort, as shown in **Supplementary Table S3**.

### Development of classification models based on all features

All 24 features from the routine blood tests were employed to train classification models based on 12 machine learning algorithms, including AdaBoost, DT, LGB, KNN, GLM, LR, RF, GB, SVM, XGB, naïve Bayes, and TabNet.

**Figure 2:**
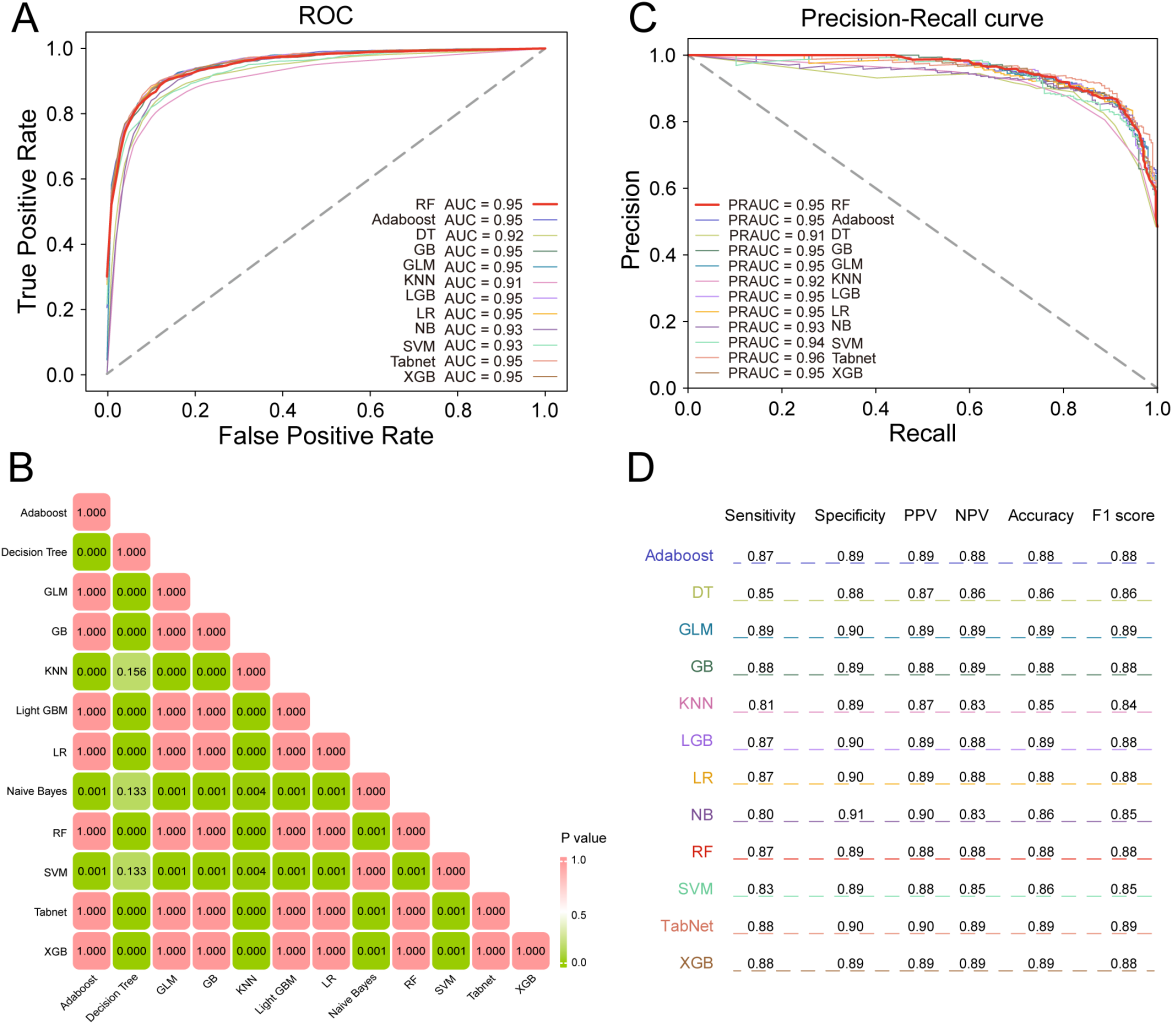
Performance of 12 machine learning models for differentiating CAP severity using routine blood indicators in the discovery cohort. **(A)** ROC curves depicting the performance of 12 machine learning models in differentiating CAP severity. **(B)** Comparison of AUC values among the 12 machine learning models using the DeLong non-parametric test. **(C)** PR curves showing the precision-recall performance of the 12 machine learning models for distinguishing CAP severity. **(D)** Detailed summary of the performance metrics for the 12 machine learning models. CAP, community-acquired pneumonia; ROC, the receiver operating characteristic curve; AUC, the area under the ROC; DT, decision tree; LGB, light gradient boosting; KNN, k-nearest neighbors; GLM, generalized linear model; LR, logistic regression; RF, random forest; GB, gradient boosting; SVM, support vector machine; XGB, extreme gradient boosting; NB, naïve Bayes; PPV, positive predictive value; NPV, negative predictive value.

In terms of the AUC (**Figure 2A**), the performance of the AdaBoost (AUC = 0.95), GB (AUC = 0.95), LGB (AUC = 0.95), LR (AUC = 0.95), RF (AUC = 0.95), TabNet (AUC = 0.95), XGB (AUC = 0.95), and GLM (AUC = 0.95) models was significantly better (P < 0.05, **Figure 2B**) than that of the other models, whose AUC values ranged from 0.91 to 0.93. Additionally, the area under the precision–recall curve (AUPRC) of the models based on the AdaBoost, GB, LGB, LR, RF, TabNet, XGB, and GLM algorithms were also higher than those of the other models (**Figure 2C**), confirming the superior performance of the former. A detailed description of the performance of these 12 models is provided in **Figure 2D** and **Supplementary Table S4**.

Given these results, the AdaBoost, GB, LGB, LR, RF, TabNet, and XGB models and the GLM were selected for inclusion in the subsequent classification model development steps.

### Identification of the final model on the basis of nine features

**Figure 3:**
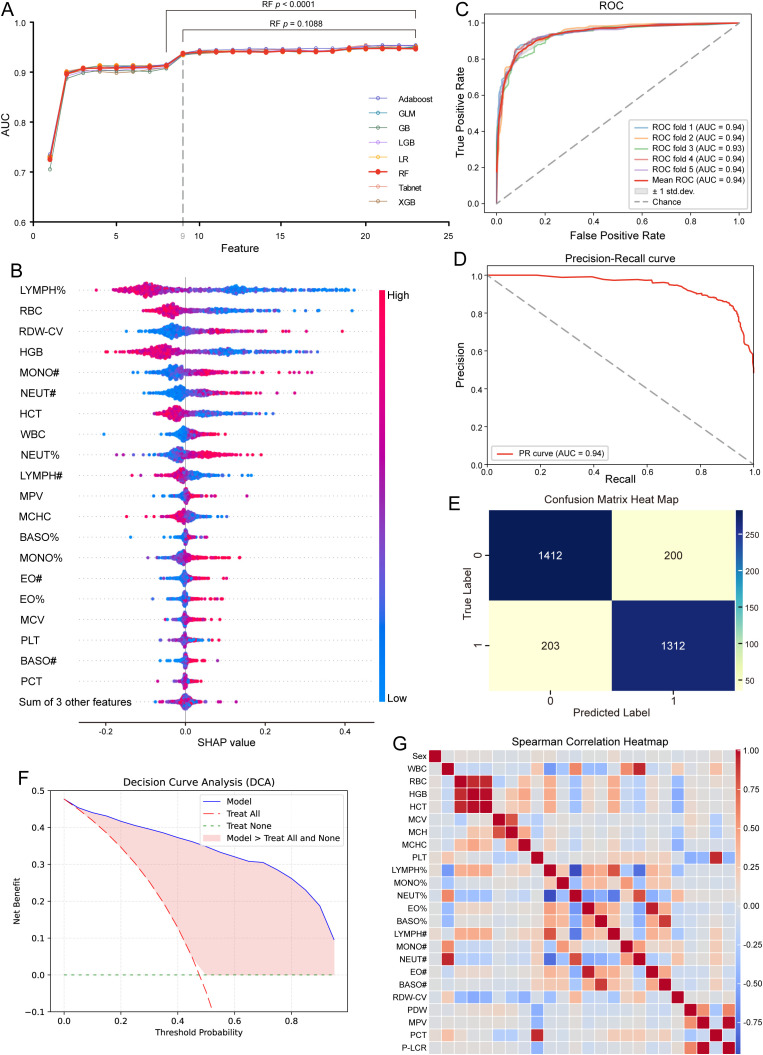
Feature characteristics and identification of the final model in the discovery cohort. **(A)** AUC values for different numbers of features selected using the SHapley Additive exPlanations (SHAP) method, along with ranking scores of all features. **(B)** SHAP summary bar plot of the RF model showing the ranking of feature importance. **(C)** ROC curves illustrating the performance of the RF machine learning model based on nine selected features in differentiating CAP severity. **(D)** PR curves displaying the precision-recall performance of the nine-feature RF model for differentiating CAP severity. **(E)** Confusion matrix heatmap showing the classification performance of the nine-feature RF model in differentiating CAP severity. **(F)** Decision curve analysis (DCA) curves evaluating the clinical utility of the nine-feature RF model. **(G)** Heatmap demonstrating the correlation among all routine blood indicators. CAP, community-acquired pneumonia; ROC, the receiver operating characteristic curve; AUC, the area under the ROC; LGB, light gradient boosting; GLM, generalized linear model; LR, logistic regression; RF, random forest; GB, gradient boosting; XGB, extreme gradient boosting; PR, precision–recall curve.

To further enhance the clinical applicability of the models, we conducted feature reduction. Features were selected using the SHAP method, and on the basis of the resulting feature importance ranking, the feature set was progressively reduced from 24 features to a single feature to determine the optimal set for maximizing predictive performance (**Figure 3A**, **Supplementary Table S5**). For the AdaBoost, GB, LGB, LR, RF, TabNet, and XGB, and the GLM, reducing the number of features for constructing the models to 10, 10, 13, 10, 9, 10, 14, and 10, respectively, resulted in no significant differences in performance with the corresponding models built from the full feature set (P > 0.05, **Supplementary Table S5**). Interestingly, the AUCs of the AdaBoost, GB, LGB, LR, RF, TabNet, and XGB models and the GLM were consistently 0.95 with the reduced feature sets.

The final model was subsequently selected according to number of features incorporated as well as the values of performance metrics such as sensitivity, specificity, PPV, NPV, accuracy, and F1 score (**Supplementary Table S6**). In this way, the RF model, which included only 9 selected features (**Figure 3B**), including LYMPH%, RDW-CV, MONO#, HGB, RBC, NEUT%, WBC, NEUT#, and HCT, and demonstrated strong performance across the abovementioned metrics, was chosen as the final model.

The 9-feature RF model achieved an AUC of 0.95 (**Figure 3C**) and an AUPRC of 0.94 (**Figure 3D**), with a PPV of 0.89, NPV of 0.88, accuracy of 0.89, and F1 score of 0.89, as detailed in **Supplementary Table S6**. A confusion matrix (**Figure 3E**) was created to visualize the performance of the RF model, showing that the sensitivity was 0.89 and the specificity was 0.90. Decision curve analysis (DCA) revealed that the final 9-feature RF model had greater clinical utility than the other models, as presented in **Figure 3F**. Finally, a heatmap (**Figure 3G**) was used to visualize the correlations among the original 24 variables.

### External validation of the final model

**Figure 4:**
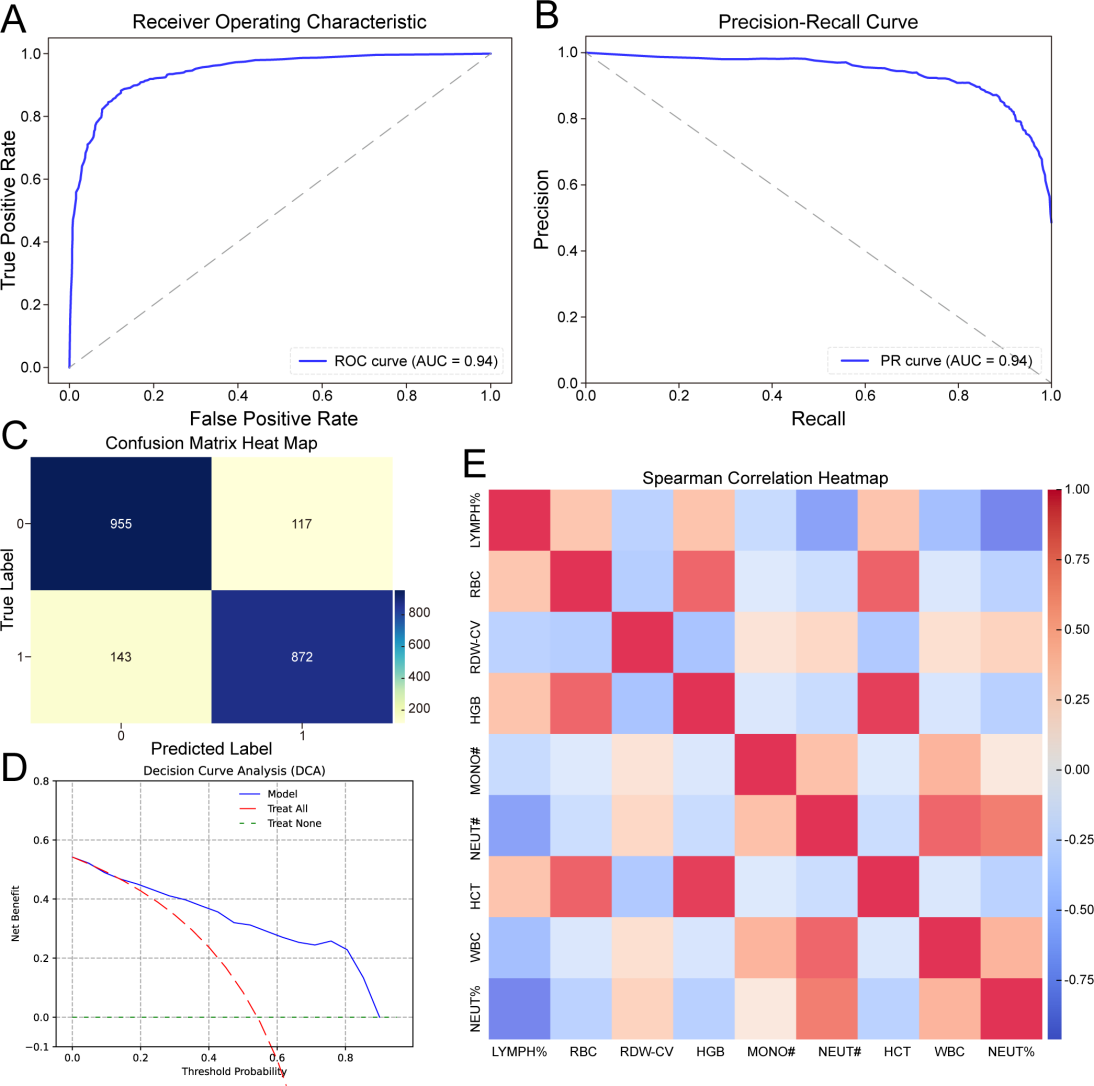
Validation of the final RF model in the independent validation cohort. **(A)** ROC curves illustrating the performance of the RF machine learning model based on nine selected features in differentiating CAP severity. **(B)** PR curves displaying the precision-recall performance of the nine-feature RF model for differentiating CAP severity. **(C)** Confusion matrix heatmap showing the classification performance of the nine-feature RF model in differentiating CAP severity. **(D)** DCA curves evaluating the clinical utility of the nine-feature RF model. **(E)** Heatmap demonstrating the correlation among all routine blood indicators. CAP, community-acquired pneumonia; ROC, the receiver operating characteristic curve; DCA, Decision curve analysis.

All models developed in the discovery cohort required validation with the independent validation cohort to assess robustness. Therefore, we employed the previously designated validation cohort to verify the performance of the 9-feature RF model. Consistent with the findings in the discovery cohort, the RF model demonstrated remarkable performance in assessing disease severity in patients with CAP. In the validation cohort, the RF model achieved an AUC of 0.95 (**Figure 4A**) and an AUPRC of 0.94 (**Figure 4B**), with a PPV of 0.88, an NPV of 0.87, an accuracy of 0.88, and an F1 score of 0.87. The confusion matrix (**Figure 4C**) also demonstrated the performance of the RF model, showing that the sensitivity was 0.86 and the specificity was 0.89.

The DCA plot in **Figure 4D** shows that the final 9-feature RF model provided consistent net benefits across the entire range of threshold probabilities, highlighting its desirable clinical utility. A heatmap (**Figure 4E**) was used to visualize the correlations among the 24 variables in the validation cohort.

Finally, we conducted a direct head-to-head comparison between our final 9-feature RF model and the PSI score. As shown in **Supplementary Figure S3A**, the PSI score achieved an AUC of 0.76, with a cutoff at risk class IV, yielding a sensitivity of 80.76% and a specificity of 80.34%. The DCA plot in **Supplementary Figure S3B** further demonstrates that the PSI score consistently provided net clinical benefits across the entire range of threshold probabilities, underscoring its practical utility in clinical decision-making. Overall, the performance of our model exceeded that of the PSI score.

### Model explanation

**Figure 5:**
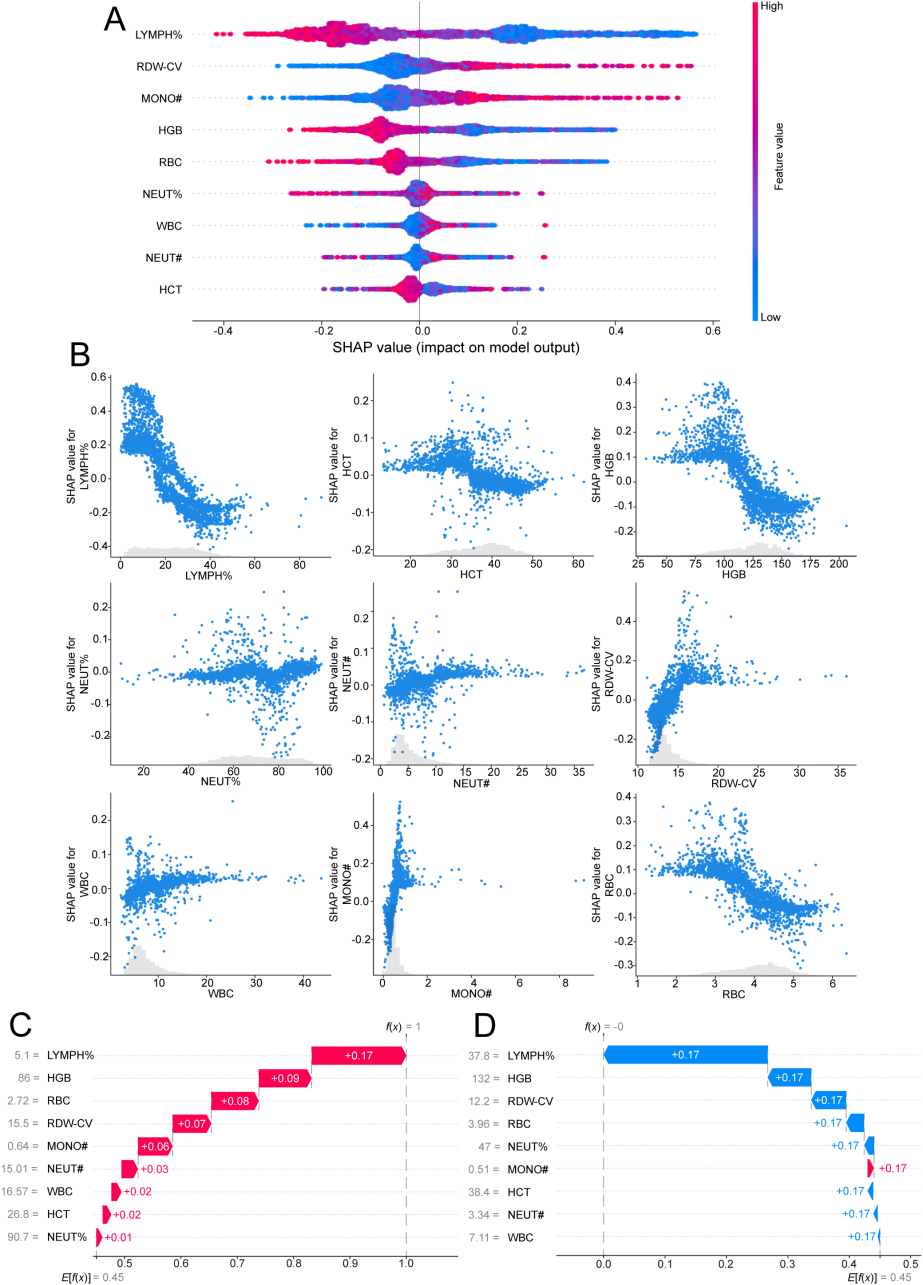
Model interpretation of the final RF model using SHAP analysis. **(A)** SHAP summary bar plot depicting the global feature importance rankings of the nine selected predictors. The features are ranked in descending order based on their mean absolute SHAP values, which reflect the average magnitude of each feature’s contribution to the model’s output across the dataset. **(B)** SHAP dependence plots demonstrating the relationship between the actual values of each of the nine selected features and their corresponding SHAP values. Each point represents an individual patient, with SHAP values greater than zero indicating a positive contribution toward predicting severe CAP. These plots reveal both the direction and magnitude of feature influence on the model’s decision-making. **(C)** Local explanation for an individual patient with mild CAP, who was misclassified by the RF model as having severe CAP with a predicted probability of 100%. The SHAP force plot (or waterfall plot, if applicable) highlights the specific features and their contributions that drove the model toward a severe classification, providing insight into potential sources of misclassification. **(D)** Local explanation for a patient with clinically confirmed severe CAP, who was incorrectly classified by the RF model as mild with a predicted probability of 0%. The plot illustrates how the combination of feature values resulted in a strong negative prediction, contrary to the actual clinical status, underscoring the importance of interpretability in understanding model errors. CAP, community-acquired pneumonia; RF, random forest; SHAP, SHapley Additive exPlanations.

To improve the interpretability and transparency of the model, the SHAP method was used to evaluate the contribution of each feature to the output of the optimal model. The global SHAP provide an overview of the model’s behavior, from which SHAP summary plots (**Figure 5A**) are generated, showing the ranked contributions of each feature on the basis of their average SHAP values in descending order.

Additionally, SHAP dependence plots were generated to aid in understanding how individual features influence the output of the RF model. **Figure 5B** illustrates the relationship between the real values and the SHAP values for the 9 selected features: SHAP values greater than zero indicate a positive class prediction, meaning that the model classifies the patient as having severe CAP.

The local SHAP demonstrates how a specific prediction is made for an individual by incorporating individual input data. **Figure 5C** illustrates the prediction for a CAP patient who was classified into the “severe” category with a probability of 100% according to the RF model. **Figure 5D** illustrates the prediction for a CAP patient who was classified into the “severe” category with a probability of 0% according to the RF model.

### Convenient application for clinical utility

**Figure 6:**
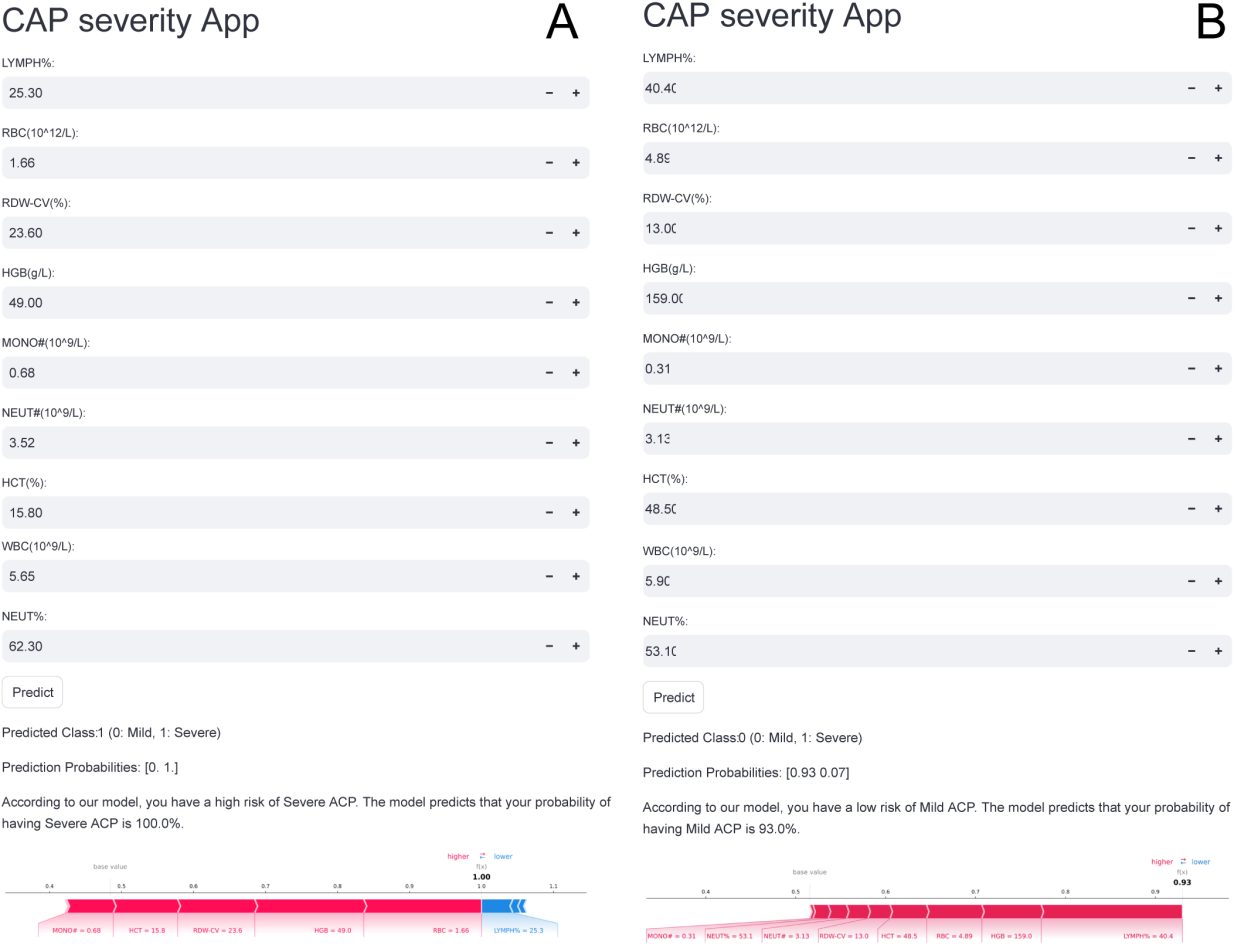
Web-based application of the prediction model for clinical utility. This figure demonstrates the user-friendly interface of the developed web application, which facilitates real-time clinical application of the final RF model. Upon entering the actual values of the nine required features, the application automatically computes the probability of severe community-acquired pneumonia (CAP). **(A)** Example of a patient predicted to be at high risk of severe CAP with a predicted probability of 100%. **(B)** Example of a patient predicted to be at high risk of mild CAP with a predicted probability of 93%. For each individual, a personalized bar plot displays the predicted probability of severe CAP. In addition, a SHAP force plot is generated to visually interpret the model’s decision for the specific patient. In the force plot, blue bars on the right represent features that contribute positively toward a severe CAP prediction, while red bars on the left represent features that drive the prediction toward mild CAP. The web-based application is publicly accessible at: https://predictionmodel-for-aki.streamlit.app.

The final RF model was integrated into a web application to enhance its practicality in clinical settings, as shown in **Figure 6**. Once the user has entered the values for the 9 features required by the model, the application automatically predicts the likelihood of severe CAP in the patient. Additionally, a force plot for the CAP patient in question is displayed, illustrating the features that influence the model toward predicting the severity of CAP. The blue features on the right are those that push the prediction toward “severe,” whereas the red features on the left push it toward “mild.” The web application is accessible online at https://predictionmodel-for-aki.streamlit.app.

## Discussion

Efficiently distinguishing the severity of CAP remains a significant challenge in clinical practice. Recent evidence has emphasized a paradigm shift from a reliance on single indicators (Morris et al., 2021) to the combined use of multiple biomarkers (Togun et al., 2020), as the latter has demonstrated greater diagnostic potential. However, identifying the optimal combination of biomarkers and establishing reliable diagnostic rules remain complex and unresolved issues. To address these challenges while minimizing testing costs, researchers have investigated leveraging routine blood indicators in conjunction with machine learning algorithms as a promising solution. Machine learning can maximize the diagnostic value of individual indicators by optimizing the use of laboratory test data. Routine blood tests, one of the most frequently performed procedures in clinical practice, provide critical insights into a patient’s immune response and inflammatory status, offering a rich source of data for diagnostic analysis (Wen et al., 2022). This integrated approach can potentially identify novel, simple, and reliable biomarkers that increase diagnostic accuracy and economical over existing methods, facilitating their incorporation into clinical practice and transforming diagnostic methodologies to improve patient outcomes.

In this study, we investigated the potential of machine learning for distinguishing mild from severe CAP through the development of 12 predictive models. Our results demonstrate that the nine-feature RF model has significant diagnostic value for effectively distinguishing mild from severe CAP, serving a promising tool for clinical application and decision-making. Several studies (Wang et al., 2017; Liu et al., 2022; Zheng et al., 2022) have recently explored new models for distinguishing the severity of CAP and predicting patient outcomes, highlighting the importance of routine laboratory indicators. For instance, the WBC count and monocyte complexity distribution width were significantly greater in the severe CAP group than in the control and common pneumonia groups (Zheng et al., 2022). Additionally, the WBC and neutrophil counts have been recognized as easily accessible biomarkers for identifying CAP patients likely to benefit from adjunctive dexamethasone treatment (Wittermans et al., 2022). These studies collectively highlight the importance of routine laboratory indicators in assessing the onset, severity, and treatment response of CAP, consistent with the findings of this study. However, these studies focused predominantly on single or small numbers of biomarkers and did not comprehensively consider differences across all routine laboratory indicators.

Several studies have demonstrated that routine blood parameters can offer valuable insights into the mechanisms underlying infectious diseases, including CAP. Crouser et al. reported that during pathogen invasion, monocytes are activated to combat the infection, resulting in a rapid increase in monocyte volume, as reflected in the monocyte distribution width. This parameter was subsequently identified as a novel and effective diagnostic indicator for sepsis (Buoro et al., 2016; Crouser et al., 2019). Taken together, these findings highlight the value of hematological parameters in diagnosing infectious diseases and suggest their potential applicability in evaluating the severity of CAP.

The RF model based on routine blood indicators demonstrated strong efficacy in distinguishing CAP severity while offering several other key advantages. First, as routine blood tests are already widely used for patient management and monitoring, no additional samples or costs are needed. Second, these tests can be completed within 30 minutes, enabling clinicians to quickly evaluate a patient’s condition. Third, the quantitative nature of the included features ensures greater objectivity and reliability than does manual observation of morphological changes. Moreover, to enhance clinical utility, the RF model has been incorporated into a web application, requiring users to input the values of only nine routine blood indicators to efficiently assess the CAP severity of their patients.

The web-based tool developed from our machine learning algorithm demonstrates strong potential for real-time clinical application. In particular, it may assist healthcare providers in promptly identifying high-risk CAP patients who are more likely to require ICU admission or closer monitoring, thereby supporting timely and optimized triage decisions. This is especially valuable in emergency departments and primary care settings, where rapid assessment is critical and access to advanced diagnostics may be limited. In such environments, the tool could function as a practical and cost-effective aid to guide decisions between inpatient and outpatient management. To establish its clinical utility, future prospective studies and implementation research are necessary to assess its effectiveness, integration into routine workflows, and overall impact on patient outcomes.

Several limitations of this study should be noted. First, while the study was conducted with a large sample size and included independent validation, the geographic scope was restricted. To ensure broader applicability, further validation in diverse populations across different regions and ethnic groups is warranted. To ensure broader applicability, further validation in diverse populations across different regions and ethnic groups is warranted. To address this limitation, we are planning future studies that will externally validate the model using data from institutions in different geographic and demographic settings. These efforts will further clarify the robustness and applicability of the model in broader populations. Second, the influence of the proposed model on clinical decision-making, including its impact on patient outcome and medication management, requires further exploration. Third, a notable limitation of this study is the inherent variability in routine blood test results, both between individuals (inter-patient variability) and within the same individual over time (intra-patient variability). Factors such as the timing of sample collection, coexisting medical conditions, hydration status, and differences in laboratory methodologies may all influence test outcomes. Although large sample sizes may help reduce the impact of random variability, this issue introduces potential noise that could affect the model’s robustness and generalizability. Future research should consider incorporating longitudinal datasets and adopting standardized sampling protocols to minimize such variability and enhance model stability. Finally, although the indicators used in this study were derived from routine laboratory tests, which increasing the practicality and clinical accessibility of the model, numerous emerging biomarkers with high diagnostic potential were not considered. Future models that integrate these novel biomarkers may achieve even greater diagnostic accuracy and utility.

## Conclusions

In conclusion, this study developed and externally validated a machine learning model based on nine routine blood test parameters (LYMPH%, RDW-CV, MONO#, HGB, RBC, NEUT%, WBC, NEUT#, and HCT) to accurately distinguish between mild and severe CAP. Among the 12 algorithms tested, the random forest model demonstrated the best performance, with consistent discriminative ability and clinical utility across large discovery and validation cohorts. By integrating these widely available hematological markers into a web-based application, this study expands the role of complete blood counts from supportive diagnostic markers to core predictors in an actionable clinical tool. These findings provide new evidence that specific blood test parameters can serve as reliable indicators for CAP severity, thereby contributing to earlier identification of patients requiring intensive management. Future prospective trials and real-world implementation studies are warranted to further validate the model’s generalizability and assess its impact on clinical decision-making, patient outcomes, and healthcare efficiency.

## Data Availability

The raw data supporting the conclusions of this article will be made available by the authors, without undue reservation.
