# Supplementary material for: A machine learning-based model for assessing community-acquired pneumonia severity using routine blood tests
Source: Front Cell Infect Microbiol. 2026 Jan 12;15:1605502. doi: 10.3389/fcimb.2025.1605502 (PMC12832646; doi:10.3389/fcimb.2025.1605502)
Supplement: Supplementary file 1 [file DataSheet1.pdf]

Supplementary Tables

Table S1. Baseline characteristics of routine laboratory indicators between mild and severe CAP in the discovery cohort

	Mild	Severe	P-value
NO.	2684	2530	
Age, Year	67.35 ± 11.45	67.29 ± 12.56	0.538
Gender, male/female	1197/1487	1102/1428	0.466
Smoking, yes/no	792/1892	745/1785	0.985
Drinking, yes/no	1009/1675	945/1585	0.880
Diabetes mellitus, yes/no	540/2144	484/2046	0.388
Hypertension, yes/no	690/1994	622/1908	0.367
Heart failure, yes/no	268/2416	288/2242	0.112
Liver disease, yes/no	114/2570	89/2441	0.197
Renal disease, yes/no	209/2475	197/2333	1.000
Cerebrovascular disease, yes/no	166/2518	170/2360	0.466
Neurological disease, yes/no	128/2556	111/2419	0.554
WBC/ (×10^{12}/L)	6.49 ± 2.19	9.62 ± 5.00	<0.001
RBC/ (×10^{12}/L)	4.47 ± 0.51	3.56 ± 0.78	<0.001
HGB/ (g·L^{-1})	136.55 ± 15.61	106.73 ± 23.67	<0.001
HCT	41.25 ± 4.47	33.05 ± 6.99	<0.001
MCV/fL	92.47 ± 5.19	93.36 ± 7.41	<0.001
MCH/Pg	30.60 ± 1.92	30.09 ± 2.63	<0.001
MCHC/ (g·L^{-1})	330.95 ± 11.75	322.32 ± 13.44	<0.001
PLT (×10^9/L)	213.02 ± 58.37	224.19 ± 101.99	<0.001
LYMPH%	29.85 ± 9.73	14.50 ± 9.78	<0.001
MONO/%	5.90 ± 1.88	6.13 ± 3.67	0.006
NEUT/%	61.61 ± 10.75	77.19 ± 12.80	<0.001
EO/%	2.36 ± 2.18	1.96 ± 3.47	<0.001
BASO/%	0.28 ± 0.23	0.22 ± 0.27	<0.001
LYMPH# (×10^9/L)	1.86 ± 0.79	1.19 ± 1.12	<0.001
MONO# (×10^9/L)	0.37 ± 0.14	0.54 ± 0.45	<0.001
NEUT# (×10^9/L)	4.10 ± 1.97	7.71 ± 4.81	<0.001
EO# (×10^9/L)	0.15 ± 0.15	0.16 ± 0.55	0.107
RDW-*CV* (%)	12.95 ± 0.97	14.63 ± 2.48	<0.001
PDW	15.83 ± 1.30	16.12 ± 0.45	<0.001
MPV/fL	9.51 ± 1.07	9.62 ± 1.24	<0.001
PCT/%	0.20 ± 0.05	0.21 ± 0.08	<0.001
PLCR/%	23.45 ± 7.41	24.07 ± 8.54	0.005
BASO# (×10^9/L)	0.02 ± 0.01	0.02 ± 0.02	0.568

Table S2. Baseline characteristics of routine laboratory indicators between mild and severe CAP in the validation cohort

	Mild	Severe	P-value
NO.	1612	1515	
Age, year	66.44 ± 12.29	67.22 ± 12.09	0.096
Gender, male/female	851/761	802/713	0.964
Smoking, yes/no	433/1179	395/1120	0.646
Drinking, yes/no	528/1084	483/1032	0.629
Diabetes mellitus, yes/no	289/1322	302/1213	0.168
Hypertension, yes/no	458/1154	431/1084	1.000
Heart failure, yes/no	189/1423	162/1353	0.392
Liver disease, yes/no	67/1545	54/1461	0.444
Renal disease, yes/no	199/1413	178/1337	0.648
Cerebrovascular disease, yes/no	170/1442	148/1367	0.510
Neurological disease, yes/no	86/1526	67/1448	0.272
WBC/（$\times 10^{12}$/L）	6.49 ± 2.18	9.68 ± 5.03	<0.001
RBC/（$\times 10^{12}$/L）	4.45 ± 0.50	3.55 ± 0.77	<0.001
HGB/（$g \cdot L^{-1}$）	136.27 ± 15.74	106.49 ± 23.51	<0.001
HCT	41.16 ± 4.51	32.98 ± 6.97	<0.001
MCV/fL	92.61 ± 5.09	93.25 ± 7.36	0.004
MCH/Pg	30.65 ± 1.88	30.06 ± 2.64	<0.001
MCHC/（$g \cdot L^{-1}$）	331.02 ± 12.19	322.36 ± 13.26	<0.001
PLT（$\times 10^{9}$/L）	212.59 ± 59.02	223.82 ± 101.55	<0.001
LYMPH%	29.78 ± 9.89	14.33 ± 9.82	<0.001
MONO/%	5.95 ± 1.94	6.08 ± 3.70	0.221
NEUT/%	61.62 ± 10.95	77.38 ± 12.92	<0.001
EO/%	2.38 ± 2.20	1.99 ± 3.67	<0.001
BASO/%	0.27 ± 0.23	0.22 ± 0.24	<0.001
LYMPH#（$\times 10^{9}$/L）	1.86 ± 0.81	1.17 ± 1.07	<0.001
MONO#（$\times 10^{9}$/L）	0.37 ± 0.14	0.55 ± 0.47	<0.001
NEUT#（$\times 10^{9}$/L）	4.09 ± 1.94	7.78 ± 4.83	<0.001
EO#（$\times 10^{9}$/L）	0.15 ± 0.16	0.17 ± 0.65	0.202
RDW-*CV*（%）	12.93 ± 0.89	14.61 ± 2.42	<0.001
PDW	15.82 ± 1.35	16.11 ± 0.44	<0.001
MPV/fL	9.52 ± 1.09	9.61 ± 1.25	0.03
PCT/%	0.20 ± 0.05	0.21 ± 0.08	0.003
PLCR/%	23.43 ± 7.56	23.99 ± 8.63	0.053
BASO#（$\times 10^{9}$/L）	0.02 ± 0.01	0.02 ± 0.02	0.351

Table S3. Performance of 12 Machine Learning Models (five-fold change) for Differentiating CAP Severity Using Routine Blood Indicators in the Discovery Cohort

	Adaboost	Decision Tree	GLM	GB	KNN	Light GBM	LR	NB	RF	SVM	TAB	XGB
**1-fold**												
**AUC**	0.95	0.93	0.96	0.95	0.91	0.95	0.95	0.93	0.95	0.93	0.95	0.96
**Sensitivity**	0.89	0.84	0.91	0.89	0.83	0.9	0.9	0.8	0.88	0.85	0.89	0.91
**Specificity**	0.9	0.9	0.89	0.88	0.88	0.89	0.89	0.91	0.89	0.89	0.91	0.89
**PPV**	0.89	0.89	0.88	0.87	0.87	0.88	0.89	0.9	0.88	0.88	0.9	0.89
**NPV**	0.9	0.86	0.92	0.89	0.85	0.91	0.9	0.83	0.89	0.87	0.9	0.91
**Accuracy**	0.89	0.87	0.90	0.88	0.85	0.89	0.9	0.86	0.89	0.87	0.9	0.9
**F1 score**	0.89	0.86	0.90	0.88	0.85	0.89	0.89	0.85	0.88	0.87	0.9	0.9
**2-fold**												
**AUC**	0.96	0.93	0.95	0.95	0.9	0.96	0.95	0.95	0.95	0.93	0.95	0.96
**Sensitivity**	0.83	0.85	0.89	0.86	0.78	0.85	0.85	0.79	0.86	0.81	0.86	0.86
**Specificity**	0.92	0.9	0.90	0.91	0.9	0.92	0.91	0.92	0.91	0.92	0.91	0.92
**PPV**	0.9	0.89	0.89	0.9	0.88	0.91	0.9	0.91	0.9	0.9	0.9	0.91
**NPV**	0.86	0.86	0.90	0.87	0.81	0.87	0.87	0.82	0.88	0.84	0.88	0.88
**Accuracy**	0.88	0.88	0.90	0.88	0.84	0.89	0.88	0.86	0.89	0.87	0.89	0.89
**F1 score**	0.87	0.87	0.89	0.88	0.83	0.88	0.87	0.84	0.88	0.86	0.88	0.89
**3-fold**												
**AUC**	0.95	0.91	0.95	0.95	0.91	0.95	0.94	0.93	0.94	0.93	0.95	0.95
**Sensitivity**	0.88	0.87	0.88	0.87	0.83	0.88	0.87	0.8	0.87	0.81	0.89	0.87
**Specificity**	0.87	0.83	0.90	0.88	0.86	0.87	0.89	0.91	0.87	0.86	0.89	0.87
**PPV**	0.86	0.83	0.89	0.87	0.85	0.87	0.89	0.9	0.86	0.85	0.88	0.87
**NPV**	0.89	0.88	0.89	0.88	0.84	0.88	0.88	0.83	0.88	0.83	0.89	0.88
**Accuracy**	0.88	0.85	0.89	0.87	0.84	0.88	0.88	0.86	0.87	0.84	0.89	0.87
**F1 score**	0.87	0.85	0.88	0.87	0.84	0.87	0.88	0.84	0.87	0.83	0.89	0.87
**4-fold**												
**AUC**	0.95	0.92	0.95	0.95	0.9	0.95	0.95	0.93	0.95	0.93	0.95	0.95
**Sensitivity**	0.87	0.84	0.88	0.89	0.81	0.87	0.86	0.83	0.88	0.83	0.88	0.87
**Specificity**	0.9	0.88	0.90	0.89	0.89	0.91	0.91	0.9	0.89	0.89	0.9	0.89
**PPV**	0.89	0.87	0.89	0.88	0.88	0.9	0.9	0.89	0.88	0.88	0.89	0.88
**NPV**	0.88	0.86	0.89	0.89	0.83	0.88	0.87	0.85	0.89	0.85	0.89	0.88
**Accuracy**	0.89	0.86	0.89	0.89	0.85	0.89	0.88	0.87	0.89	0.86	0.89	0.88
**F1 score**	0.88	0.86	0.88	0.88	0.84	0.89	0.88	0.86	0.88	0.85	0.89	0.88
**5-fold**												
**AUC**	0.95	0.91	0.95	0.96	0.92	0.95	0.95	0.94	0.95	0.94	0.96	0.95
**Sensitivity**	0.88	0.84	0.87	0.89	0.81	0.87	0.88	0.79	0.86	0.83	0.89	0.88
**Specificity**	0.89	0.87	0.91	0.89	0.89	0.89	0.89	0.92	0.89	0.88	0.91	0.89

**PPV**	0.88	0.86	0.90	0.88	0.88	0.88	0.88	0.9	0.88	0.87	0.9	0.89
**NPV**	0.89	0.86	0.88	0.89	0.83	0.88	0.89	0.82	0.87	0.85	0.9	0.89
**Accuracy**	0.88	0.86	0.89	0.89	0.85	0.88	0.88	0.86	0.88	0.86	0.9	0.89
**F1 score**	0.88	0.85	0.88	0.88	0.84	0.88	0.88	0.84	0.87	0.85	0.9	0.89

Table S5. Performance of reduced feature-based Machine Learning Models (five-fold change) for Differentiating CAP Severity Using Routine Blood Indicators in the Discovery Cohort

	**10-Adaboost**	**10-GB**	**13-Light GBM**	**10-LR**	**9-RF**	**10-Tabnet**	**14-XGB**	**10-GLM**
**Average**								
**AUC**	0.95	0.95	0.95	0.95	0.95	0.95	0.95	0.95
**Sensitivity**	0.87	0.88	0.87	0.87	0.89	0.88	0.87	0.87
**Specificity**	0.89	0.89	0.89	0.90	0.90	0.90	0.89	0.87
**PPV**	0.89	0.88	0.88	0.89	0.89	0.89	0.88	0.87
**NPV**	0.88	0.88	0.88	0.88	0.88	0.89	0.88	0.87
**Accuracy**	0.88	0.88	0.88	0.88	0.89	0.89	0.88	0.86
**F1 score**	0.88	0.88	0.88	0.88	0.89	0.89	0.87	0.88
**1-fold**								
**AUC**	0.95	0.95	0.95	0.95	0.95	0.94	0.95	0.95
**Sensitivity**	0.88	0.89	0.89	0.90	0.89	0.87	0.89	0.87
**Specificity**	0.87	0.89	0.88	0.90	0.88	0.90	0.89	0.86
**PPV**	0.86	0.88	0.88	0.89	0.87	0.89	0.88	0.86
**NPV**	0.89	0.89	0.90	0.90	0.89	0.88	0.90	0.88
**Accuracy**	0.88	0.89	0.89	0.90	0.89	0.89	0.89	0.87
**F1 score**	0.87	0.88	0.88	0.90	0.88	0.88	0.89	0.87
**2-fold**								
**AUC**	0.96	0.95	0.96	0.95	0.95	0.96	0.95	0.95
**Sensitivity**	0.84	0.86	0.85	0.84	0.89	0.90	0.84	0.87
**Specificity**	0.93	0.91	0.92	0.90	0.90	0.91	0.90	0.87
**PPV**	0.92	0.90	0.90	0.89	0.90	0.91	0.89	0.87
**NPV**	0.86	0.87	0.87	0.86	0.88	0.90	0.86	0.88
**Accuracy**	0.89	0.88	0.88	0.87	0.89	0.91	0.87	0.87
**F1 score**	0.88	0.88	0.88	0.86	0.89	0.90	0.87	0.88
**3-fold**								
**AUC**	0.95	0.94	0.95	0.94	0.94	0.95	0.94	0.94
**Sensitivity**	0.86	0.88	0.88	0.86	0.86	0.89	0.87	0.87
**Specificity**	0.89	0.86	0.86	0.89	0.84	0.90	0.85	0.86
**PPV**	0.88	0.85	0.86	0.88	0.84	0.89	0.85	0.87
**NPV**	0.87	0.88	0.88	0.87	0.86	0.90	0.87	0.87
**Accuracy**	0.87	0.87	0.87	0.88	0.85	0.90	0.86	0.86
**F1 score**	0.87	0.87	0.87	0.87	0.86	0.89	0.86	0.88
**4-fold**								
**AUC**	0.95	0.94	0.95	0.95	0.96	0.94	0.94	0.94

**Sensitivity**	0.87	0.87	0.87	0.85	0.90	0.86	0.87	0.87
**Specificity**	0.89	0.91	0.90	0.91	0.90	0.89	0.90	0.86
**PPV**	0.88	0.90	0.89	0.90	0.89	0.88	0.89	0.87
**NPV**	0.88	0.88	0.88	0.87	0.89	0.88	0.88	0.87
**Accuracy**	0.88	0.89	0.88	0.88	0.91	0.88	0.88	0.86
**F1 score**	0.88	0.88	0.88	0.87	0.89	0.87	0.88	0.88
**5-fold**								
**AUC**	0.95	0.95	0.95	0.95	0.95	0.94	0.95	0.95
**Sensitivity**	0.87	0.89	0.87	0.88	0.88	0.88	0.88	0.88
**Specificity**	0.90	0.89	0.90	0.89	0.87	0.89	0.89	0.87
**PPV**	0.89	0.88	0.89	0.88	0.86	0.88	0.88	0.88
**NPV**	0.88	0.89	0.88	0.89	0.89	0.89	0.89	0.87
**Accuracy**	0.89	0.89	0.89	0.89	0.88	0.88	0.88	0.86
**F1 score**	0.88	0.89	0.88	0.88	0.88	0.88	0.88	0.89

Supplementary Figures

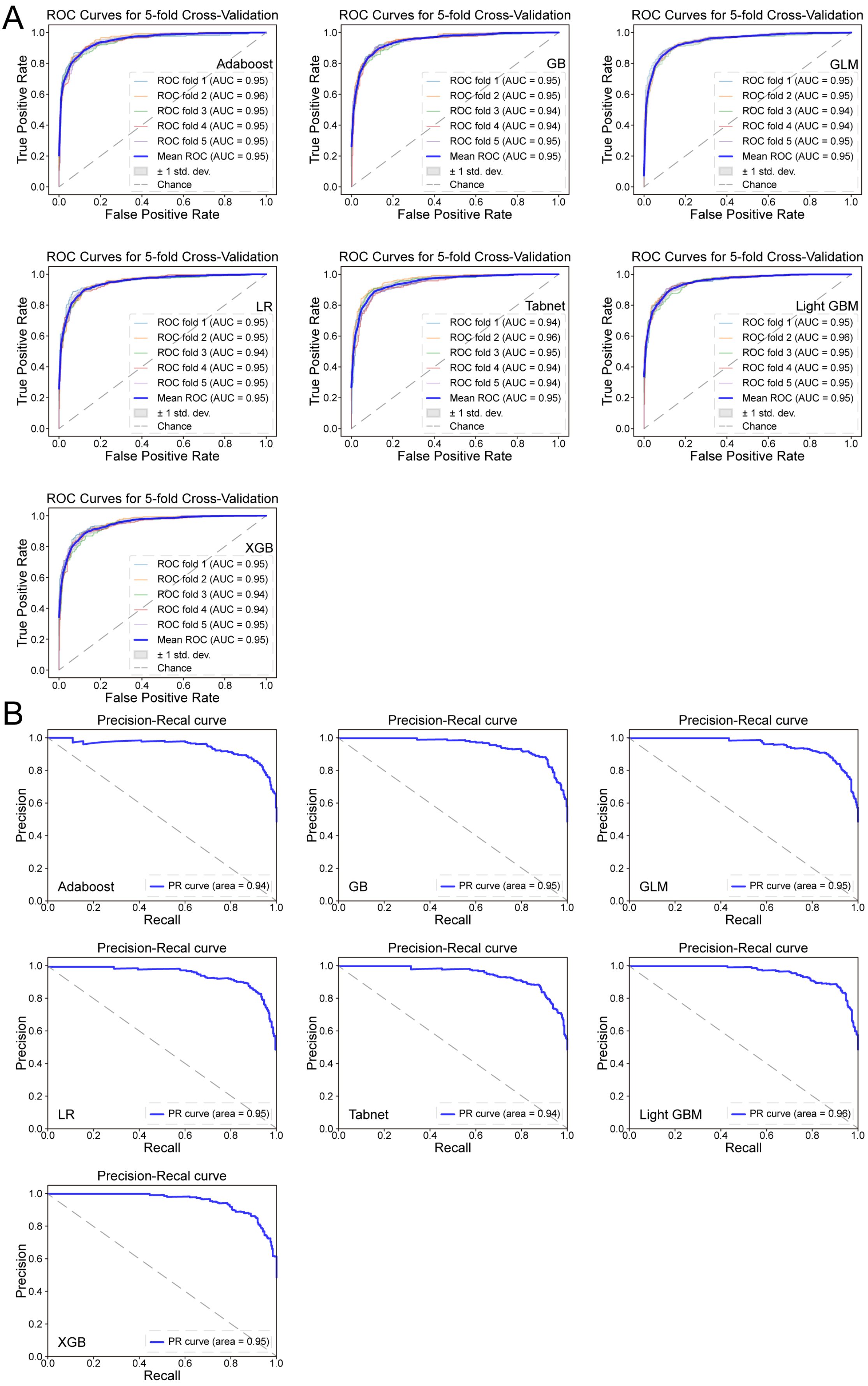

Figure S1. Performance of reduced feature-based Machine Learning Models for Differentiating CAP Severity Using Routine Blood Indicators in the Discovery Cohort

A: ROC curves illustrating the performance of these machine learning model based on the selected features in differentiating CAP severity.

D: PR curves displaying the precision-recall performance of these machine learning model based on the selected features for differentiating CAP severity.

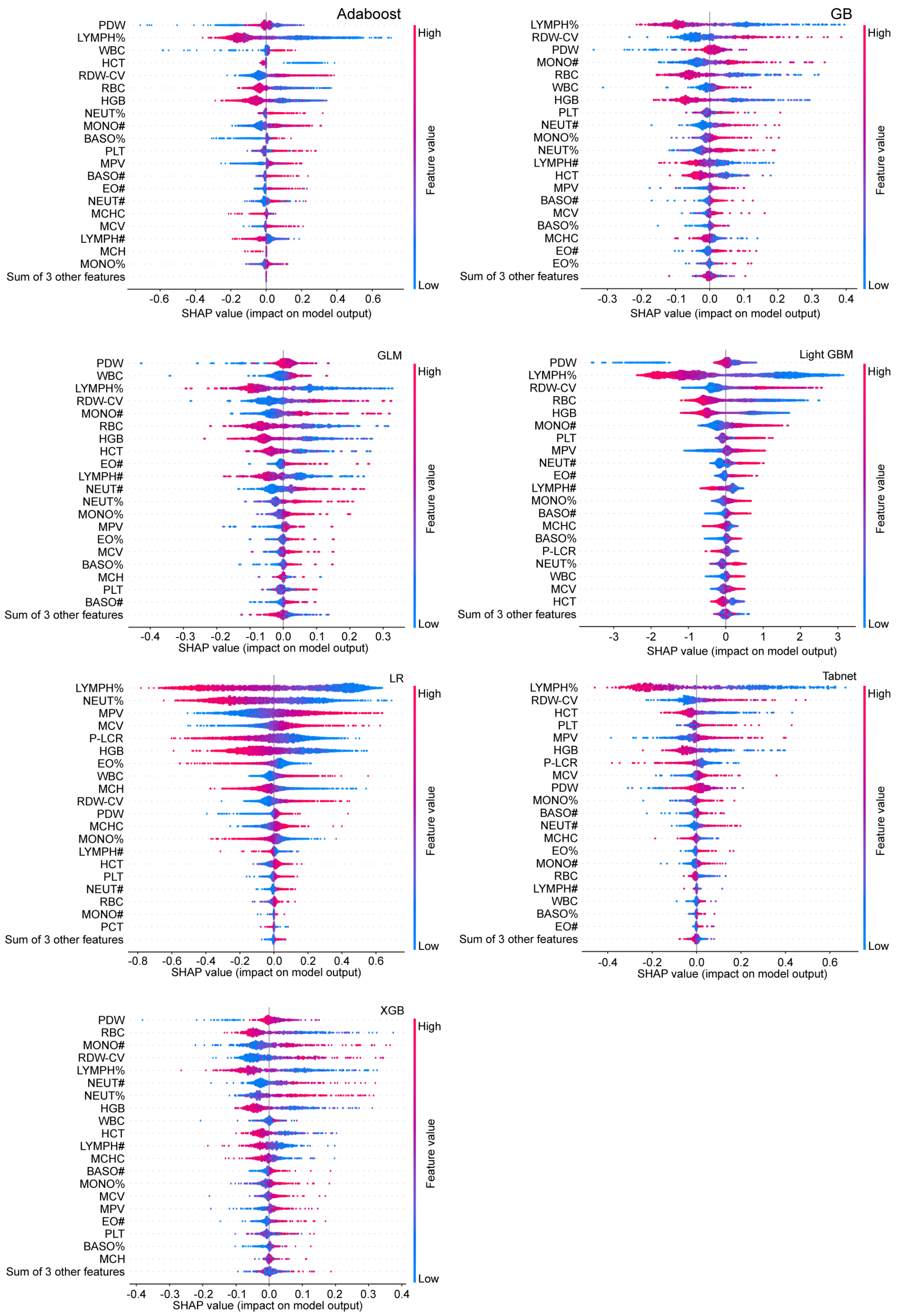

Figure S2. SHAP summary bar plot of the Machine Learning model showing the ranking of the feature importance.

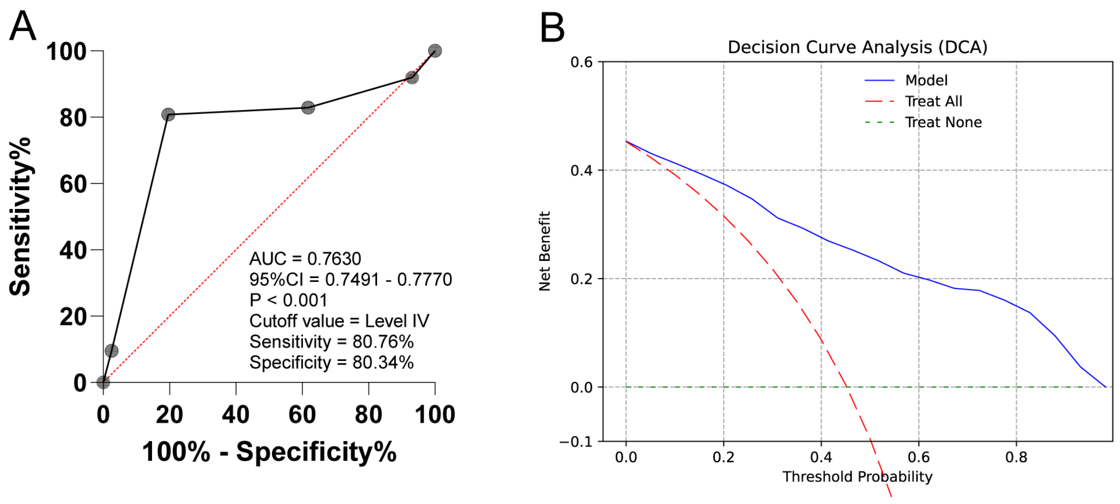

Figure S3. Performance of PSI scores for Differentiating CAP Severity

A: ROC curves depicting the performance of PSI scores for differentiating CAP severity.

B: Decision curve analysis (DCA) curves evaluating the clinical utility of the PSI scores for differentiating CAP severity.
